# Editorial: XXXIII SIMGBM Congress 2019 - Antimicrobials and Host-Pathogen Interactions

**DOI:** 10.3389/fmicb.2021.672517

**Published:** 2021-04-07

**Authors:** Flavia Marinelli, Pietro Alifano, Paolo Landini, Paolo Visca

**Affiliations:** ^1^Dipartimento di Biotecnologie e Scienze della Vita, Università degli Studi dell'Insubria, Varese, Italy; ^2^Dipartimento di Scienze e Tecnologie Biologiche ed Ambientali, Università del Salento, Lecce, Italy; ^3^Dipartimento di Bioscienze, Università degli Studi di Milano Statale, Milano, Italy; ^4^Dipartimento di Scienze, Università degli Studi Roma Tre, Roma, Italy

**Keywords:** antibiotics, bacteria, fungi, bacterial communication, host-pathogen interactions, pathogenesis, antimicrobial resistome, cell-to-cell signaling

This Research Topic (RT) is intended to provide a collection of selected contributions in the broad area “Antimicrobials and Host-Pathogen Interactions” from the participants of “Microbiology 2019” congress organized by the Italian Society for General Microbiology and Microbial Biotechnology (SIMGBM, www.simgbm.it), which was held in Florence, Italy, on June 19–22, 2019. The congress was attended by 224 scientists from all over the world, and contributions relevant to this RT were collected from presentations in the following congress sessions: (i) Antibiotic resistome: where do antibiotic resistance genes come from? (ii) New antimicrobial strategies in the post-antibiotic era; (iii) Intercellular communication in host-pathogen interactions; (iv) New approaches to unravel fungal-host interactions; (v) Bacterial cell surface and signaling. The unifying concept of this RT originates from the increasing awareness that antimicrobial resistance (AMR) is a complex problem which should be addressed by a multifocal approach. Fundamental microbiological investigations in this direction involve understanding of the flow of AMR genes from the environment to human and animal pathogens, the development of new drugs to tackle AMR, and the discovery of new druggable targets to impair microbial growth and/or pathogenicity.

## Antimicrobial Resistance

AMR has initially been observed and studied in hospitals, since the massive use of antimicrobials in clinical settings was considered as its major driving force. However, the origin of AMR is currently considered to be a natural phenomenon that predates the antimicrobial use by humans (D'Costa et al., 2011). AMR genes constitute the so-called resistome, which is defined as the environmental pool of genes conferring either intrinsic or acquired AMR (Surette and Wright, 2017). In their review, Hernando-Amado et al. highlighted that the dissemination of AMR genes is not limited only to the environments characterized by the exposure to high antimicrobial concentrations. Sub-inhibitory antimicrobial levels and the presence of heavy metals promote horizontal gene transfer (HGT), thus increasing further environmental dissemination of AMR genes. In environmental bacteria, AMR genes are often co-localized with mobile genetic elements (MGEs) (e.g., transposons, plasmids, and integrons) that facilitate their intra and inter species transfer. In the case of transmissible infections, a socioecological global approach is needed to preserve the health of the environment, since an increasing crowding of human and food animal populations, the consequent environmental pollution and misuse of drugs favors pathogen transmission both at the local and global levels.

A possible environmental source of AMR genes can be identified among the soil-dwelling bacteria, which produce antibiotics and need protection from them (Binda et al., 2014). Recently, a massive sequencing effort of antibiotic-producer genomes revealed that AMR genes are often clustered together with antibiotic biosynthesis genes and respond to the same regulatory circuits controlling both the production and resistance. In the minireview by Yushchuk et al., this association was confirmed for the environmental actinobacteria, which produce glycopeptide antibiotics and belong to the *Amycolatopsis, Actinoplanes, Nonomuraea*, and *Streptomyces* genera. Glycopeptide antibiotics such as vancomycin, teicoplanin, and dalbavancin are clinically important drugs for treating severe hospital-acquired infections caused by multiresistant Gram-positive bacteria (Marcone et al., 2018). A recent pioneering work on the phylogenetic reconstruction of glycopeptide biosynthetic gene clusters has paved the way to track the simultaneous evolution of glycopeptide synthesis and resistance genes, and the recruitment and combination of the latter in pathogens (Waglechner et al., 2019).

One of the most common and difficult-to-treat healthcare-associated infections is due to methicillin- resistant *Staphylococcus aureus* (MRSA). Worryingly, an increasing number of MRSA is becoming resistant to other antibiotics, such as β-lactams, carbapenems, glycopeptides, and to the drug of last resort, daptomycin (Shariati et al., 2020). Daptomycin is a lipopeptide, which is naturally produced by the soil saprotroph *Streptomyces roseosporus* (Miao et al., 2005). Both the mode of action and mechanism of resistance toward daptomycin have not completely elucidated yet (Miller et al., 2016). In the paper of Cafiso et al., the authors interrogated the multifactorial nature of MRSA resistance to daptomycin by comparing a clinical daptomycin-resistant isolate vs. its isogenic daptomycin-sensitive strain and by using comparative genomics and late-growth phase transcriptomics. Both strains were isolated from the same patient undergoing daptomycin therapy in hospital during a period of several months. The daptomycin-resistant strain acquired a diverse set of genomic changes under antibiotic selection, which included mutations in genes encoding cell membrane proteins and transcriptional regulators. Transcriptomic changes were found in cell wall and cell membrane organization genes, in the autolytic system, and in primary metabolism genes switching it toward fermentation.

The paper by Almebairik et al. described the role of MGEs in the evolution of *Staphylococcus epidermidis* from a commensal to a nosocomial diseases-causing agent. *S. epidermidis* and other coagulase negative staphylococci represent a leading cause of both bloodstream and skin and soft tissue infections in nosocomial settings (Otto, 2009). Whole genome sequencing of 58 *S. epidermidis* bacteremic isolates revealed the prevalence and evolution of arginine catabolic mobile element (ACME) and copper and mercury resistance (COMER)-like mobile elements, frequently associated with staphylococcal cassette chromosome *mec* (SCCmec) forming composite genetic elements. Considerable variations of ACME occurred even between isolates belonging to the same ACME and sequence types (ST). Similarly, the authors could detect previously undescribed COMER-like elements showing high stability in 11 *S. epidermidis* isolates belonging to ST2, i.e., one of the two major hospital-adapted drug-resistant lineages. The difference in stability at the genomic level correlated well with the higher excision frequency found for the SCCmec elements in ACME-containing isolates compared to COMER-like element-containing isolates, suggesting that the presence of COMER-like elements represents the most prominent accessory genome feature determining the epidemiological success of ST2 *S. epidermidis* lineage.

## Novel Targets and Molecules

Notwithstanding the AMR crisis, the number of novel antibiotic classes being introduced into the clinical practice since 2000 has been very limited, and daptomycin is one of the few antimicrobial drugs launched since the 2000s (Payne et al., 2007). This situation is even more worrisome for Gram-negative pathogens, where treatment options are extremely limited (Van Camp et al., 2020). Consequently, new druggable targets and active molecules are urgently needed to treat infections caused by Gram-negative bacteria.

Mellini et al. exploited the possibility of targeting the PqsR quorum sensing (QS) regulatory protein to suppress *Pseudomonas aeruginosa* virulence. QS is involved in an intercellular communication process, and it is a global regulator of expression of key virulence traits. Therefore, PqsR inhibitors could be potential anti-virulence agents (Ellermann and Sperandio, 2020). Starting from the virtual screening of a large library of FDA-approved drugs, five compounds were selected based on *in silico* prediction of their binding affinity to PqsR. One of these compounds reduced the expression of *P. aeruginosa* virulence traits *in vitro*, highlighting the potential of virtual screening and drug repositioning in the selection of new FDA-approved anti-virulence drugs (Konreddy et al., 2019). Structural knowledge of bacterial proteins implicated in vital functions or host interactions, combined with computational predictions of their potential ligands, made the strategy described by Mellini et al. a promising approach to the rapid identification of potential inhibitors.

A target-based approach was also used by Chiarelli et al. to identify small molecules inhibiting the function of the FtsZ protein in *Burkholderia cenocepacia*. Since FtsZ is an essential and extremely conserved component of the bacterial divisome, it represents an attractive target for antimicrobials (Haranahalli et al., 2016). The growing clinical importance of *B. cenocapacia*, especially among cystic fibrosis patients, and its intrinsic antibiotic resistance pose an urgent need to discover new effective drugs against this pathogen (Scoffone et al., 2017). More than 50 derivatives of the benzothiadiazole compound C109 were tested *in vitro* using purified *B. cenocapacia* FtsZ as a target, with the IC_50_ range between 3 and 100 μM. The growth inhibiting activity was further investigated by conventional susceptibility tests with *B. cenocepacia, P. aeruginosa* and *S. aureus*. Although C109 showed some limitations for *B. cenocepacia* growth inhibition, being the substrate for efflux pumps and modification enzymes, it was endowed with a potent antibacterial activity against *S. aureus* and displayed relatively low cytotoxicity. The study highlighted a rationale for choosing a broadly conserved target, such as FtsZ, in the successful screening campaign of bacterial growth inhibitors.

The paper by Moura et al. pointed to the bacterial outer membrane as a promising target for the development of novel antibiotics against Gram-negative bacteria. Thanatin, a 21-residue inducible cationic defense peptide isolated from the hemipteran insect *Podisus maculiventris*, has a unique mode of action targeting the lipopolysaccharide (LPS) transport (Lpt) system by binding to the N-terminal β-strand of LptA and causing defects in membrane assembly (Vetterli et al., 2018). The authors implemented the Bacterial Adenylate Cyclase Two-Hybrid (BACTH) system to probe *in vivo* the Lpt interactome in the periplasm, and found that thanatin targets both LptC–LptA and LptA–LptA interactions, with a greater inhibitory effect on the former. The disruption of LptC–LptA interaction was confirmed *in vitro* by nuclear magnetic resonance and surface plasmon resonance experiments, while LPS analysis in crude extracts demonstrated that in cells treated with thanatin, LPS accumulates at the periplasmic side of the inner membrane, where it is decorated with colanic acid, in a dead-end reaction diagnostic of defects in the Lpt system (Sperandeo et al., 2011).

Tuberculosis (TB) is the leading cause of morbidity and mortality due to a single infectious agent. About one-quarter of the world population has the latent form of TB, thus representing a large and persistent reservoir for active infection. Due to this, the emergence of multidrug resistant *Mycobacterium tuberculosis* strains represents a major public health threat worldwide (Koch et al., 2018). The thienopyrimidine TP053 is a promising new antitubercular lead, with a potent activity against both replicating and non-replicating *M. tuberculosis* cells (Albesa-Jové et al., 2014). Its mode of action involves the metabolic activation to a nitric oxide (NO)-releasing intermediate by mycobacterial mycothiol-dependent reductase Mrx2. To gain further insights into the mechanism of action of TP053, Mori et al. isolated *M. tuberculosis* TP053-resistant mutants, which carried a L240V substitution in Rv0579, which is still an uncharacterized protein. Protein co-purification experiments demonstrated that activated TP053 binds not only to Mrx2, but also to Rv0579, suggesting that a direct interaction between the activated prodrug and Rv0579 is implicated in resistance. Intriguingly, TP053 treatment caused the up-regulation of Rv0582, which encodes the VapC26 toxin in wild-type *M. tuberculosis*, but not in the Rv0579 resistant mutant, suggesting that Rv0579 could be involved in the regulation of VapC26 expression. While the actual role of Rv0579 in *M. tuberculosis* physiology remains still elusive, the data reported in this paper point to an involvement of Rv0579 in RNA metabolism and expression of the mycobacterial toxin anti-toxin system.

## Microbial Biofilms

Many bacterial pathogens are able to form biofilms during infection, which render them less susceptible to antimicrobials and to the action of the host immune system, thus contributing to antibiotic therapy failure. Microbial biofilms are notoriously more tolerant to antibiotics than planktonic cells (Wolfmeier et al., 2018). However, results from *ex vivo* analysis of pathological samples indicate that there is a continuum between the biofilm and planktonic lifestyles, since suspended bacterial aggregates are a major form of bacterial communities in chronic infection sites (Cai, 2020).

Bidossi et al. collected sinovial fluids from joint infections and confirmed the presence of fibrin-embedded *S. aureus* and *Staphylococcus lugdunensis* planktonic aggregates in their samples. Clumps were also generated *in vitro*, when bacteria were grown in synovial fluid, and this resulted in an up to 32-fold increase of recalcitrance to antibiotic treatments. Moreover, exposure to synovial fluid caused morphological alterations of staphylococcal biofilm structures on a titanium surface and increased the overall biofilm volume. This *ex vivo* evidence uncovers the mechanism of staphylococcal adoptation to the growth in synovial fluids implemented in the aggregated form. This finding highlights the importance of antibiotic susceptibility testing under conditions which should be closer to *in vivo* situations, in order to configure a successful therapy of joint infection.

The introduction of a foreign body (e.g., an implantable device) is known to trigger bacterial adhesion and biofilm formation as some bacteria have a preferred tropism for abiotic surfaces (Arciola et al., 2018). Several bacterial infections are linked to the utilization of medical percutaneous devices, such as catheters or electrical drivelines. By perforating the skin to reach internal organs, these devices often serve as entry points for opportunistic pathogens found on our skin, such as *S. aureus*. Bolle et al. developed a model of skin broken by a mock electrical driveline and performed experimental infection experiments with *S. aureus* to evaluate the efficacy of human skin equivalent. They showed that *S. aureus* migration into the wound, biofilm formation and tissue damage could be mitigated by human skin equivalent, particularly when seeded with fibroblasts.

Although most tissue and medical device-associated infections are caused by a single pathogen, an increasing number of polymicrobial infections have recently been reported (Rodrigues et al., 2019). The microorganisms involved are thought to coexist in synergistic relations within the biofilm environment, thus resulting in enhanced pathogenicity, virulence, and resistance to antimicrobials. Ceresa et al. investigated the effect of bacterial-sourced biosurfactants as inhibitors of clinically relevant fungal/bacterial dual-species biofilms, which are formed on polystyrene plates and on medical-grade silicons. They tested a rhamnolipid produced by *P. aeruginosa*, a lipopeptide from *Bacillus subtilis* and a sophorolipid from *Candida albicans* by measuring their effect on biofilm-forming *C. albicans* and *Staphylococcus* spp. The rhamnolipid was the most effective, reducing the metabolic activity and biomass of biofilms by >90% within 72 h. It was active when used adsorbed on silicone, and it was not-cytotoxic, supporting its potential in controlling device-associated infections.

Endophytic bacteria leaving in association with different plant tissues can be exploited as a source of new antimicrobials. With this aim, Castronovo et al., investigated the microbiota of the essential oil-producing *Origanum vulgare*, by culture dependent and independent approaches, and next-generation sequence analysis. Bacterial isolates from different anatomic parts of the plant were tested for antibiotic resistance, antagonistic interactions, and antimicrobial activity against multidrug resistant pathogens. Nearly 50% of them were found to release compounds active against at least one bacterial pathogen, with a marked selectivity against Gram-positive rather than Gram-negative bacteria, substantiating the idea that novel molecules can be discovered by mining microbe-plant interactions.

## Host-Pathogen and Symbiotic Interactions

There is constant communication between hosts and bacteria that can lead to two alternative scenarios: either tolerance and/or resistance of the bacterial species within the host that leads to quiescent host habitation, or disease characterized by host tissue damage and rapid replication of the bacteria (Casadevall and Pirofski, 2000). Opportunistic pathogens, often in the context of multifactorial diseases, such as *P. aeruginosa, Helicobacter pylori*, and adherent-invasive *Escherichia coli*, play a role in exacerbation of cystic fibrosis, stomach cancer, and Crohn's disease, respectively.

Cavinato et al. addressed the role of superoxide dismutases (SOD) in *P. aeruginosa* survival in macrophages. In intracellular pathogens, SODs can counteract the bactericidal effect of ROS generated in macrophages by the NOX2 NADPH oxidase (Lam et al., 2010). The authors showed, counterintuitively, that knocking out *sodB*, which is one of the two genes encoding periplasmatic SODs in *P. aeruginosa*, leads to the enhanced bacterial survival in macrophages, apparently due to the reduced turnover of ${\text{O}}_{2}^{-}$ into the even more reactive oxygen species H_2_O_2_. However, the *sodB* mutant showed reduced long-term intracellular survival, due to its inability to inhibit autophagy in the macrophage. This work highlights the specialized roles of SOD isoenzymes in response to the macrophage oxidative burst, thus providing survival mechanisms for different stages of bacterial infection.

Soluri et al. probed the yet unclear connection between *H. pylori* and oncogenesis, and pursued the hypothesis that some *H. pylori* antigens may trigger an overly inflammatory reaction, leading to gastric ulcers, which, in turn, can progress to cancer. Using a phage display technology, the authors constructed a phage library encompassing the whole *H. pylori* genome, and searched for proteins that would strongly react with the sera of patients affected by *H. pylori-*related oncological pathologies. This approach led to the identification of the CagY/Cag7 protein, an already known *H. pylori* virulence factor (Rohde et al., 2003), as a specific antigen in cancer patients. These results point to a possible role of this protein (or for antibodies against it) as a potential marker for early detection of stomach cancer.

Adherent-invasive *E. coli* (AIEC) are overrepresented in the microbiota of Crohn's disease patients, where they play a pivotal role in triggering chronic inflammation typical of the disease, especially due to their ability to survive intracellularly. Fanelli et al. investigated the role of genes encoding efflux pumps involved in multidrug resistance, monitoring their expression during bacterial invasion of either intestinal epithelial cells or macrophages. The authors showed that several efflux pump genes are induced during intracellular infection and that one efflux pump in particular (MdtE) contributed to AIEC survival in human macrophages. In addition to improving our understanding of AIEC pathogenesis mechanisms, these results suggest that efflux pumps might be targets for drugs alleviating AIEC-induced inflammation in Crohn's disease.

Obligate intracellular bacteria represent a useful model to investigate bacterial interactions with host cells, and *Rickettsiaceae* provide a paradigmatic example of bacterial endosymbiosis with a number of eukaryotic organisms (protists, green algae, and metazoan) (Salje, 2021). Pasqualetti et al. investigated the newly described “*Candidatus Megaira polyxenophila*,” an endosymbiont of eukaryotes living in marine, brackish, or freshwater habitats. By analyzing two phylogenetically close species of the freshwater ciliate *Paramecium* that are naturally infected by “*Ca. M. polyxenophila*,” the authors found that both *Paramecium* species have growth advantages in the presence of *Ca. M. polyxenophila*. This advantage is reproduced at every tested salinity condition, although the fitness effects of the endosymbiosis vary between the two *Paramecium* species and environmental conditions. They observed the loss of the endosymbiont after prolonged exposure to higher salinity levels, and this explains why “*Ca. M. polyxenophila*” is more abundant in freshwater than in marine habitats. Infection experiments with these bacteria had so far been unsuccessful under laboratory conditions, so this works provides the very first insights into possible effects of *Ca. M. polyxenophila* on its host.

## Concluding Remarks

As reflected in articles of this RT, antimicrobial resistance, new antibacterial drugs and the mechanisms of host-pathogen interactions remain hot topics, which attracted many contributions and provided a rather faithful snapshot of the quality and the variety of subjects presented at the XXXIII SIMGBM Congress 2019. Progress in these fields is rapidly advancing, and we hope that we will able to discuss them at the next SIMGBM meeting. Our meeting, planned for 2020, has been put on hold due to the Covid-19 pandemic. We hope to host a meeting “in the flesh” on 15th−18th September 2021, to be held in Cagliari, on the beautiful island of Sardinia, if the public health emergency allows it. We wish to encourage our readers to stay tuned to the news on this conference, and we consider it as a new “kick-off” in attending a meeting after a long time of unavoidable restrictions imposed on social and scientific events.

## Author Contributions

FM, PA, PL, and PV gave the same contribution to the drafting of the article and have made a substantial, direct, intellectual contribution to the work. All authors contributed to the article and approved the submitted version.

## Conflict of Interest

The authors declare that the research was conducted in the absence of any commercial or financial relationships that could be construed as a potential conflict of interest.
